# Peripheral Nerve Injury Is Associated with Chronic, Reversible Changes in Global DNA Methylation in the Mouse Prefrontal Cortex

**DOI:** 10.1371/journal.pone.0055259

**Published:** 2013-01-28

**Authors:** Maral Tajerian, Sebastian Alvarado, Magali Millecamps, Pascal Vachon, Cecilia Crosby, M. Catherine Bushnell, Moshe Szyf, Laura S. Stone

**Affiliations:** 1 Alan Edwards Centre for Research on Pain, Faculty of Medicine, McGill University, Montreal, Canada; 2 Department of Anesthesiology, McGill University, Montreal, Canada; 3 Department of Neurology and Neurosurgery, McGill University, Montreal, Canada; 4 Department of Pharmacology and Therapeutics, McGill University, Montreal, Canada; 5 Sackler Program for Epigenetics and Psychobiology, McGill University, Montreal, Canada; 6 Faculty of Dentistry, McGill University, Montreal, Quebec, Canada; 7 University of Montreal, Department of Veterinary Biomedicine, St-Hyacinthe, Quebec, Canada; University of Kentucky Medical Center, United States of America

## Abstract

Changes in brain structure and cortical function are associated with many chronic pain conditions including low back pain and fibromyalgia. The magnitude of these changes correlates with the duration and/or the intensity of chronic pain. Most studies report changes in common areas involved in pain modulation, including the prefrontal cortex (PFC), and pain-related pathological changes in the PFC can be reversed with effective treatment. While the mechanisms underlying these changes are unknown, they must be dynamically regulated. Epigenetic modulation of gene expression in response to experience and environment is reversible and dynamic. Epigenetic modulation by DNA methylation is associated with abnormal behavior and pathological gene expression in the central nervous system. DNA methylation might also be involved in mediating the pathologies associated with chronic pain in the brain. We therefore tested a) whether alterations in DNA methylation are found in the brain long after chronic neuropathic pain is induced in the periphery using the spared nerve injury modal and b) whether these injury-associated changes are reversible by interventions that reverse the pathologies associated with chronic pain. Six months following peripheral nerve injury, abnormal sensory thresholds and increased anxiety were accompanied by decreased global methylation in the PFC and the amygdala but not in the visual cortex or the thalamus. Environmental enrichment attenuated nerve injury-induced hypersensitivity and reversed the changes in global PFC methylation. Furthermore, global PFC methylation correlated with mechanical and thermal sensitivity in neuropathic mice. In summary, induction of chronic pain by peripheral nerve injury is associated with epigenetic changes in the brain. These changes are detected long after the original injury, at a long distance from the site of injury and are reversible with environmental manipulation. Changes in brain structure and cortical function that are associated with chronic pain conditions may therefore be mediated by epigenetic mechanisms.

## Introduction

Chronic pain is associated with changes in brain structure and function. Multiple studies have now reported decreased brain grey matter and abnormal cortical function associated with chronic pain, and the magnitude of these changes may be related to the duration and the intensity of chronic pain. While changes in some brain regions are associated with specific pain conditions, many studies report changes in common areas involved in pain modulation, including the prefrontal cortex (PFC) (for reviews see [1], [2]. Interestingly, the PFC has also been implicated in depression and anxiety, both of which are co-morbid with chronic pain.

Chronic pain induces and actively maintains pathological changes in the PFC: The induction of nerve injury in normal rats results in the development of hypersensitivity to sensory stimuli and in decreased grey matter in the PFC several months post-injury [3]. Furthermore, reducing chronic pain in humans reverses pain-related changes in PFC structure and function [4], [5]. However, the mechanisms underlying chronic pain-induced neuroplasticity are currently not understood.

Epigenetic modulation of gene expression in response to experience and environmental changes is both dynamic and reversible. Covalent modification of DNA by methylation is a critical epigenetic mechanism resulting in altered gene expression. The recognition of the role of DNA methylation in human disease started in oncology but now extents to other disciplines including neurological disorders, and modulation by DNA methylation is associated with abnormal behavior and pathological gene expression in the central nervous system (CNS). For example, adverse environments early in life result in stable pathological changes in methylation and gene function in the adult [6], [7], [8], [9], [10] that are reversible with epigenetic drugs [11], [12]. A plausible working hypothesis is that long-term changes in DNA methylation in the brain embed signals from transient injury or other exposures to alter genome function in the brain, resulting in either the chronification of pain or contributing to the co-morbid pathologies associated with chronic pain. If this hypothesis is correct, then DNA methylation changes in the brain should be detectable long after exposure to the initial peripheral injury that triggered the chronic pain.

The objectives of the current study were a) to determine if a peripheral nerve injury that triggers long-term, persistent behavioural signs of neuropathic pain and a decrease in grey matter in the PFC several months post-injury [4] also triggers region-specific changes in DNA methylation in the brain that can be detected long after the initial injury and b) to determine whether these changes are sensitive to an environmental manipulation that attenuates pain. The primary findings are a) 5–6 months following peripheral nerve injury, alterations in global DNA methylation are observed in the PFC and amydala but not in the visual cortex or thalamus, b) environmental enrichment reduces both behavioural signs of neuropathic pain and pathological changes in PFC global methylation, and c) PFC global methylation significantly correlates with the severity of mechanical and cold sensitivity. Long-term alterations in DNA methylation could therefore provide a molecular substrate for chronic pain-related alterations in the CNS, forming a “memory trace” for pain in the brain that can be targeted therapeutically.

## Materials and Methods

### Animals

Two cohorts of 8–10 week-old male CD1 mice (Charles River, St-Constant, QC, Canada) were used. Animals were housed in ventilated polycarbonate cages and received water and rodent diet (Teklad Rodent Diet 2020X) ad libitum.

**Figure 1:**
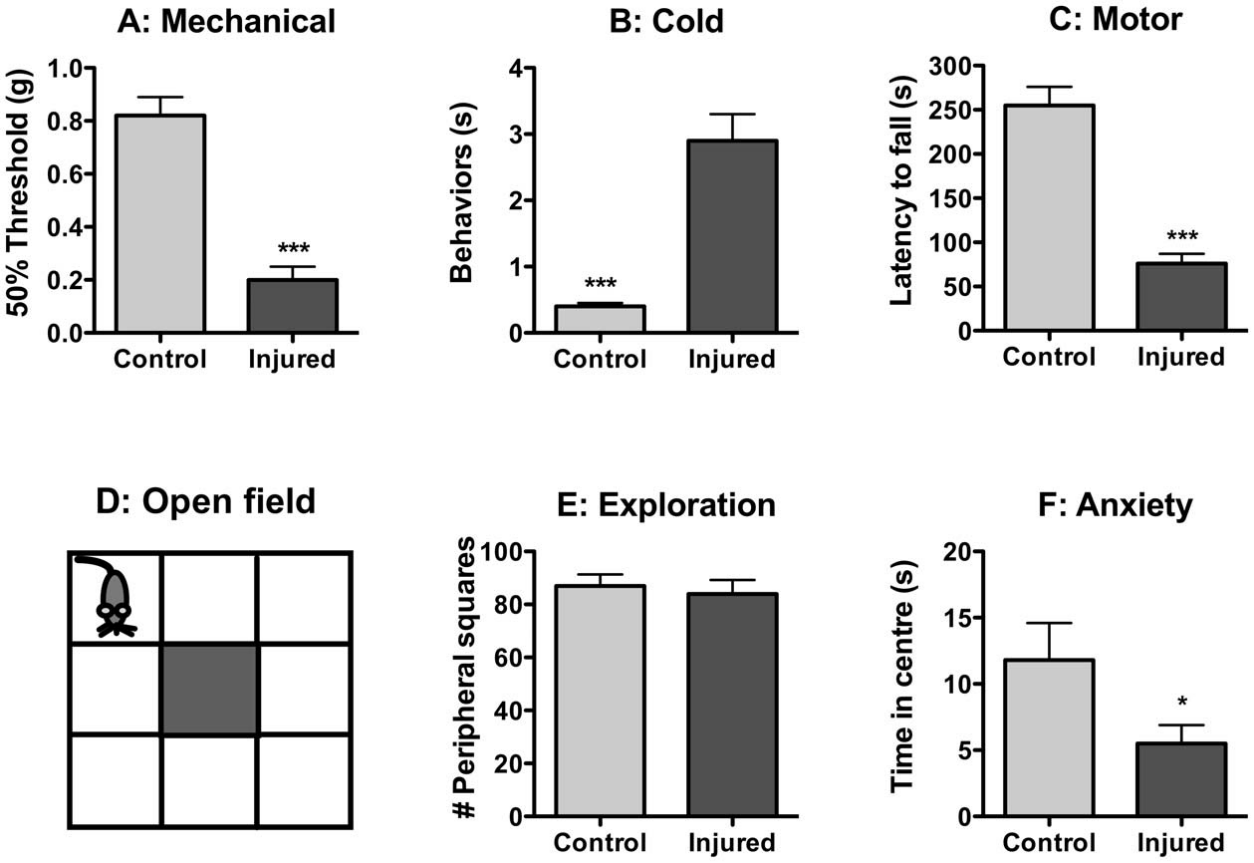
Behavioral Signs of Neuropathic Pain Six Months following Nerve Injury. Nerve injured mice show a decrease in mechanical thresholds (**A**) and an increase in acetone-evoked behaviors, indicative of cold sensitivity (**B**) on the hindpaw, measured by the von Frey filament test and the acetone tests, respectively. In addition, these mice show signs of motor dysfunction, measured by the rotarod assay (**C**). In the open field assay (**D**), neuropathic mice do not differ from control mice in overall levels of spontaneous activity, measured by the number of peripheral squares covered in the open field (**e**). However, they spent less time spent in the central square of the open field, indicative of anxiety-like behavior (**f**). * = p<0.05, *** = p<0.0001, n = 10/group, error bars indicate S.E.M.

**Figure 2:**
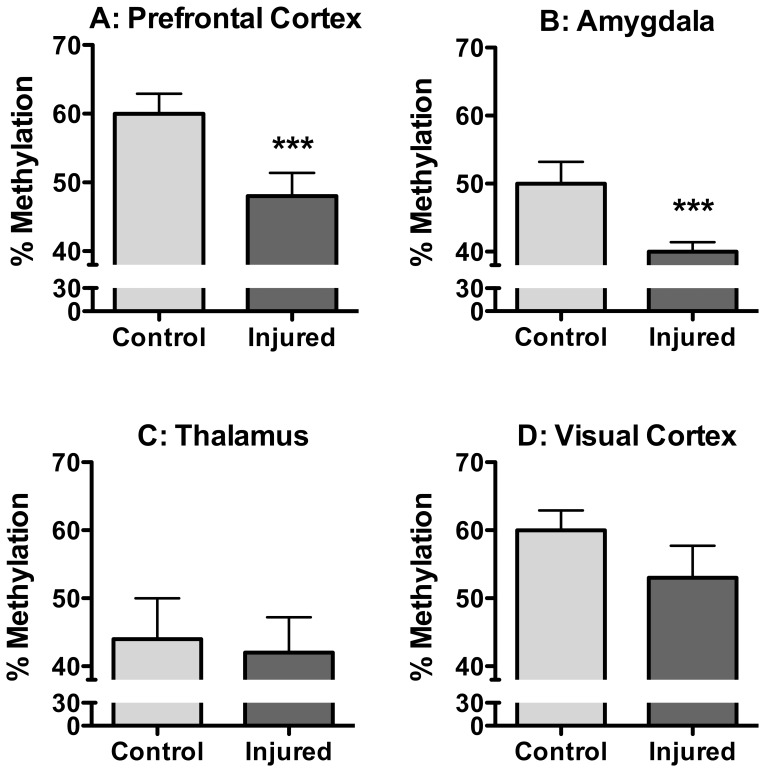
Global Methylation in the CNS Six Months following Nerve Injury. Nerve injured mice show a decrease in global methylation in the PFC (**a**) and amygdala (**b**) six months after the induction of peripheral neuropathy. No such changes were observed in the thalamus (**c**) or visual cortex (**d**). *** = p<0.0001, n = 8–10/group, error bars indicate S.E.M.

Animals in the standard environment (Figures 1&2) were housed in groups of 3–4 with a cardboard hut and cotton nesting material. In contrast, the enriched environment consisted of three mice/cage, a home cage running wheel mounted on a plastic hut (Mouse Igloo® with Fast-Trac running wheel, http://www.bio-serv.com), and marbles. In the impoverished environment, each animal was housed singly in an individual cage in the absence of a running wheel, marbles or any other forms of enrichment. All other factors including diet, bedding, access to water and light-dark cycle were identical.

All experiments were approved by the Animal Care Committee at McGill University, and conformed to the ethical guidelines of the Canadian Council on Animal Care and the guidelines of the Committee for Research and Ethical Issues of the International Association for the Study of Pain published in PAIN, 16 (1983) 109–110. All surgery was performed under isoflurane anesthesia, and all efforts were made to minimize suffering.

### Induction of Nerve Injury

Neuropathy was induced using the spared nerve injury model. Under deep anesthesia, an incision was made on the lateral surface of the thigh through the muscle, exposing the three terminal branches of the sciatic nerve: the sural, common peroneal and tibial nerves. The common peroneal and the tibial nerves were tightly ligated with 6.0 silk (Ethicon) and sectioned distal to the ligation. The sural nerve was left intact. Sham surgery involved exposing the nerve without damaging it [13].

### Behavioral Assessment

**Figure 3:**
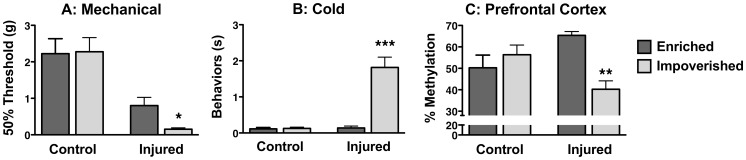
Environmental Enrichment Reverses Nerve Injury-Induced Neuropathic Pain and Pathological Changes in DNA Methylation in the Prefrontal Cortex. Three months following nerve injury and the establishment of chronic neuropathic pain, mice were subjected to an enriched or an impoverished environment for an additional two months. Environmental enrichment resulted in decreases in mechanical (**A**) and cold sensitivity (**B**) in nerve-injured animals towards control values. (**C**) These changes in cutaneous hypersensitivity following environmental enrichment were accompanied by reversal of the nerve injury-induced decreases in global methylation in the impoverished group such that global methylation was no longer different from control values (1-way ANOVA, F_(3,21)_ = 4.545, p = 0.013, tukeys multiple comparison test). * = p<0.05, ** = p<0.01,*** = p<0.001; control, enriched vs. impoverished or injured, enriched vs. impoverished; n = 5–8/group, error bars indicate S.E.M.

**Figure 4:**
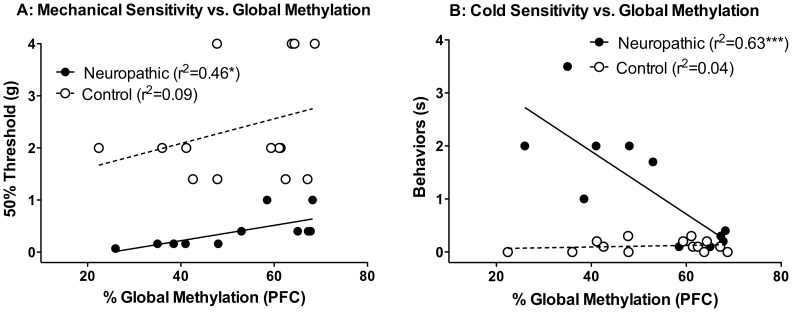
Global Methylation in the Prefrontal Cortex Correlates with the Magnitude of Nerve Injury-Induced Hypersensitivity. Correlation analysis was performed on the data from Figure 3. Significant correlations were observed between % global methylation and hypersensitivity to mechanical (**a**) and cold stimuli (**b**), in neuropathic but not control mice. * = p<0.05, ** = p<0.01.

All animals underwent baseline behavioral assessments immediately prior to surgery and no differences were observed between groups (data not shown). The first cohort were then re-assessed six months following nerve injury or sham surgery control (Figures 1 and 2). In the environmental study (Figures 3 and 4), the presence of nerve injury-induced hypersensitivity was confirmed three months following surgery when the environmental manipulations were implemented and again two months after environmental change.

#### Mechanical Sensitivity

Calibrated monofilaments (Stoelting Co., Wood Dale, IL) were applied to the plantar surface of the hindpaw and the 50% threshold to withdraw (grams) was calculated as previously described [14]. The stimulus intensity ranged from 0.008 g to 4 g.

#### Cold Sensitivity

A modified version of the acetone drop test [15] was used: total duration of acetone-evoked behaviors (flinching, licking or biting) was measured for 1 minute after acetone (∼25 µl) was applied to the plantar surface of the hindpaw with the aid of a blunt needle attached to a syringe.

#### Motor Function

The accelerating rotarod assay was used (IITC Life Science Inc., Woodland Hills, CA) with the mouse adapter [16]. The task includes a speed ramp from 0 to 30 rpm over 60 s, followed by an additional 240 s at the maximal speed.

#### Overall Activity

Mice were individually placed individually into the centre of a transparent open field (26×26 cm^2^) in a quiet room illuminated with white light and their spontaneous behavior was videotaped. The floor of the apparatus was equally divided into nine squares. The total number of squares visited in a 5 minute period was assessed. An animal must fully enter the square for it to be considered as visited. Since each square is similar in size to an average mouse (∼8–10 cm), the number of squares visited serves as a proxy measure for general motor activity.

#### Anxiety-like behavior

The same open field was used with the primary measure being the time spent in the central square during the 5 minute task [17].

### Environmental Manipulation

Three months after injury or sham surgery, the presence of neuropathic pain was confirmed and mice were randomly assigned to one of four groups: injured with enriched environment, injured with impoverished environment, sham with enriched environment, sham with impoverished environment. The standard, enriched and impoverished environments are described above. Mice were then re-tested 2 months following environmental manipulation and tissue was collected.

### Tissue Extraction

In the first study (Figures 1 and 2), animals were sacrificed 6 months after nerve injury or sham surgery by decapitation following isoflurane anesthesia. In the enrichment experiment (Figures 3 and 4), animals were sacrificed 5 months after nerve injury or sham, of which the final 2 months were spent in enriched or impoverished environments. Anatomical regions were defined according to the stereotaxic coordinates (rostral–caudal, medial–lateral and dorsal–ventral from bregma) by Paxinos and Franklin [18]. The prefrontal cortex (right and left; +1 to +3, −1 to +1, 0 to −2.5), amygdala (right and left; −1 to −3, ±4 to ±1.5, −4 to −6), thalamus (0 to −3, −2 to + 2, −2.5 to −4.25), and visual cortex (right and left; −2 to −4, −3 to +3, 0 to 2) were extracted, frozen on dry ice and stored at −80 C until use.

### DNA Extraction

Tissue was homogenized and incubated in DNA extraction buffer (500 µl) containing proteinase K (20 µl; 20 mg/ml; Roche, Basel, Switzerland) at 50°C for 12 h. Samples were treated with RNAase A (50 U/mg; 30 min; Roche) and phenol: chloroform (1∶1) added. After phase separation, ethanol (95%) was added to precipitate the DNA. The DNA pellet was then washed and redissolved in ddH_2_O.

### Luminometric Methylation Assay (LUMA)

LUMA is a high throughput assay used to determine genomic global DNA methylation. The LUMA method used in our study is a modification described by Karimi et al. [19], [20]. LUMA involves the digestion of genomic DNA by methylation sensitive (HPAII) or insensitive (MSPI) restriction enzymes in combination with an internal control restriction enzyme (EcoRI) to normalize the DNA input. Both HpaII and MspI restriction enzymes recognize and cleave 5′-CCGG-3′ sequences producing 5′-CG overhangs, whereas EcoRI recognizes and cleaves 5′-GAATTC-3′ sequences and produces 5′-AATT overhangs. The extent of cleavage is determined by a bioluminetric polymerase extension assay based on a four-step pyrosequencing reaction.

Samples were incubated (37°C, 4 h) and then heat inactivated (80°C, 20 min). Digested genomic DNA (15 µl) was mixed with pyrosequencing annealing buffer (15 µl; Qiagen, Toronto, ON, Canada). Samples were transferred to 24-well pyrosequencing plates for sequencing (PyroMark 24; Biotage, Uppsala, Sweden). The nucleotide dispensation order used was based on Pilsner et al. [21]. Peak heights for C and A represent the HpaII and MspI cuts (methylation) and EcoRI (input DNA), respectively. The formula to calculate % genomic methylation is: 1-[(HpaII (C)/EcoRI (A)/MspI (C)/EcoRI (A)]×100. All samples were run in triplicate.

### Statistical Analysis

All data are expressed as mean ± standard error of the mean (SEM). Comparisons between injured and control groups (Figures 1 and 2) or between enriched vs. impoverished (Figures 3A,B) were performed using 2-tailed, unpaired student's t-test. The data in Figure 3C was analyzed by one-way ANOVA followed by Tukeys test for multiple comparisons. Significance was set at P<0.05. Welch's correction was used when the assumption of equal variances was not met. The degree of co-variation between mechanical or cold sensitivity and global methylation was determined by calculating Pearson's coefficient since our data followed a Gaussian distribution. Statistical analysis was undertaken using Prism (GraphPad Software Inc, San Diego, California).

## Results

### Behavioral signs of neuropathic pain, motor impairment and anxiety six months following peripheral nerve injury

Chronic, persistent changes in cutaneous sensitivity to both mechanical and cold stimuli were detected six months following nerve injury. Injured mice displayed decreased mechanical thresholds (**Figure 1A**) and increased acetone-evoked behaviors (**Figure 1B**). Animals with nerve injury were significantly impaired in motor capacity in the rotarod assay (**Figure 1C**).

Animals with neuropathy exhibited signs of anxiety in the open field assay six months after injury (**Figure 1D**). Despite the fact that injured animals visited the same number of peripheral squares as control animals (**Figure 1E**), they spent significantly less time in the central square compared to control mice (**Figure 1F**).

### Nerve injury was associated with decreased global methylation in the prefrontal cortex and amygdala, but not the thalamus or visual cortex, six months following injury

Peripheral nerve injury resulted in a decrease in global methylation in the prefrontal cortex (**Figure 2A**) and the amygdala (**Figure 2B**) that was detected 6 months after injury. No significant changes were observed in the thalamus (**Figure 2C**) or visual cortex (**Figure 2D**). Since there were no significant differences in global methylation between right and left brain structures, the results reported here are the average of the two sides.

### Environmental enrichment reverses nerve injury-induced chronic pain and abnormal global methylation in the prefrontal cortex

Three months after peripheral nerve injury, a second set of mice were removed from standard housing and placed in either an enriched or an impoverished environment for two additional months. The enriched environment was associated with decreased nerve injury-induced hypersensitivity to mechanical and cold stimuli compared to the impoverished environment (**Figures 3A,B**). This was accompanied by the recovery of nerve injury-induced reductions in global methylation in the PFC to normal, control levels (**Figure 3C**).

### Correlation between global methylation in the prefrontal cortex and hypersensitivity to cutaneous stimuli

Analysis of the data from Figure 3 revealed significant correlations between the magnitude of both mechanical (**Figure 4A**) and cold (**Figure 4B**) hypersensitivity and global methylation in the PFC in injured, but not in control animals. Specifically, following environmental manipulation, nerve-injured animals with higher global % methylation in the PFC had reduced hypersensitivity to mechanical and thermal stimuli.

## Discussion

### DNA Methylation and Chronic Neuropathic Pain

Epigenetic mechanisms triggered by injury have been hypothesized to participate in mediating the lasting changes in the CNS associated with chronic pain [22]. To date, most of the work related to chronic pain has focused on the role of histone acetylation/deacetylation [23], mainly using pharmacological tools [24], [25], [26], [27], with few reports of changes in DNA methylation in chronic pain conditions [28], [29], [30], [31]. In this study, a mouse model of neuropathic pain following peripheral nerve injury was used to test the hypothesis that ongoing, chronic painful neuropathy induces changes in global DNA methylation in the brain.

Our data show decreases in global DNA methylation in the PFC and amygdala six months following a peripheral nerve injury in the hindlimb. This is consistent with many of the comorbidities that develop when pain has transitioned from being acute to chronic, such as chronic-pain associated depression [32]. Furthermore, these global changes were region-specific; similar effects were not observed in the thalamus or the visual cortex, even though the former receives direct input from nociceptive neurons. It is important to note that regions that did not show global changes may still undergo changes in DNA methylation at the individual gene level that are not detectable by a global methylation assay such as the LUMA. However, the fact that alterations were observed in the PFC and amygdala shows a strong link between nerve injury-induced hypersensitivity and changes in DNA methylation in the brain and provides a potential link between injury, chronic pain and co-morbidities such as cognitive dysfunction, depression and anxiety.

The magnitude of the nerve injury-associated changes in global methylation in the PFC from 60% to 48% suggests that the changes are broad and affect wide parts of the genome. It is estimated that the mouse genome contains ∼20 million CpG sites, the targets for DNA methylation. The LUMA assay used in this study is sensitive to ∼1.5 million of these sites. Therefore, a decrease of 12% in this assay corresponds to a minimum estimated demethylation of ∼180,000 CpG sites following nerve injury, a number predicted to alter the expression of hundreds of individual genes [33].

Global methylation is an indicator of the overall state of the DNA methylation machinery and has long-range consequences on genome function and organization [34], [35], [36]. Recent data suggests that the landscape of altered DNA methylation in other pathologies such as cancer spans thousands of genes [37] and intergenic regions [38]. Programming of DNA methylation encompasses both global changes in genome methylation and gene-specific changes that target discreet regulatory regions, thus affecting gene expression. Changes in global DNA methylation state affect high-level organization of genome function [39]. These changes produce lasting effects on the regulation of the transcriptome and higher order chromatin folding [40] and are capable of affecting many aspects of cell function. The population of methylated CpG sites in gene promoters and known regulatory regions constitutes only a small fraction of global DNA methylation. Given the magnitude of the pathological changes in DNA methylation observed in this study, they must therefore also involve regions in the genome beyond individual gene promoters and gene regulatory sequences. Indeed, demethylation/methylation of all known promoters and regulatory regions in the genome would in itself not have a significant impact on global DNA methylation.

### Dynamic Mechanisms Mediating Chronic Pain

In this study, the epigenetic changes were attenuated by a behavioral intervention. Environmental enrichment reversed nerve injury-related reductions in global DNA methylation in the PFC and reduced hypersensitivity to mechanical and cold stimuli. Furthermore, the % global PFC methylation co-varied with the severity of neuropathic pain. It is currently unclear why similar correlations were not observed in the uninjured, control mice. While it is also not clear whether it is the enrichment itself or the pain attenuation that is mediating the reversal of hypo-methylation in the PFC, data from the enrichment experiment nonetheless suggests that the methylation changes in the brain are dynamic and reversible by a behavioral intervention. Regardless, the particularly relevant since, in human patients with low back pain, both pain duration and intensity has been related to reduced grey matter in the PFC [41], and the magnitude of pain reduction following treatment correlated with corresponding increases in the thickness and normalization of functional activity in the PFC [4]. We therefore speculate that the regulation of global methylation such as described here may contribute to the dynamic changes in cortical structure and function observed in human chronic pain patients.

### Distance from the Time and Site of Injury

The main finding emphasized in this manuscript is the long-range effects of peripheral nerve injury on the mouse methylome. Equally interesting is the observation that these methylation changes occur at a site distant from the original injury. While epigenetic changes have been reported in the dorsal root ganglia and spinal cord following persistent pain states [30], [31], here we focused on higher-order processing centers in the brain. Interestingly, in the study by Wang et al., decreasing global DNA methylation in the spinal cord resulted in attenuation of pain symptoms in the first two weeks following chronic constriction of the sciatic nerve in rats; this is the opposite of what we would predict in the PFC [30]. Thus, the directionality and consequences of changes in global DNA methylation in chronic pain may be region-specific (spinal vs. supraspinal), species-specific (rat vs. mouse), may vary by type of injury or may vary as a function of chronicity (2 weeks vs. 6 months). Each of these possible explanations has potential clinical implications, additional studies are needed to further explore this discrepancy.

Pain is more than mere nociception; according to the International Association for the Study of Pain (IASP), pain is defined as “…an unpleasant sensory and emotional experience associated with actual or potential tissue damage, or described in terms of such damage” [42]. It is therefore crucial that we study the effects of chronic pain in areas that are involved in perception and emotional processing, such as the PFC and amygdala. Our data draws attention to the nature of chronic pain as a complex phenomenon: it is associated with higher order behavioral co-morbidities beyond changes in nociceptive thresholds, and it encompass a wide range of conditions that make chronic pain a disease that is difficult to understand and to treat.

### Limitations and Future Directions

The current data is consistent with the working hypothesis that DNA methylation is involved in chronic pain: a peripheral injury that leads to chronic pain triggers changes in global DNA methylation. However, it does not define a causal relationship between DNA methylation in the brain and chronic pain or its associated pathologies nor does it establish a relationship between these changes in DNA methylation and changes in gene expression. Future studies could address the causal relationships by evaluating the effects of pharmacological or environmental modulation of DNA methylation on pain threshold.

Although our data shows that environmental enrichment returned nerve injury-related changes in global DNA methylation to control levels, it is possible that a certain populations of individual gene promoters maintained their differentially methylated state. Future studies incorporating comprehensive, high throughput analysis of changes in DNA methylation and their effect on the expression of individual genes in chronic pain conditions are needed. Such studies are currently underway in our laboratory.

Our study does not distinguish between the effects of nerve injury from those of ongoing chronic pain and its comorbidities. It is possible that the observed supraspinal changes are due to other effects of the nerve injury itself such as motor impairment instead of being a consequence of living with chronic pain. Finally, the current study does not examine the time-course of global methylation changes, instead focusing on the long-term effects of peripheral neuropathy on the brain. Further studies are needed to determine how long after nerve injury changes in global DNA methylation develop and if they contribute to or are the result of pain chronification.

Our data is consistent with two alternative but not mutually exclusive hypotheses regarding the involvement of DNA methylation in chronic pain. First, DNA methylation might mediate the effects of peripheral nerve injury on chronic pain by altering epigenetic programming in the brain and inducing the central phenotypes associated with chronic pain. Second, chronic pain might induce the DNA methylation changes, which in turn trigger the downstream pathologies that accompany chronic pain. It is also possible that DNA methylation is involved in both processes. These questions need to be addressed in future studies. Understanding the mechanisms underlying the transition from transient injury to chronic pain as well as the mechanisms mediating the impact of chronic pain on mental and physical health are questions of prime significance. Our study shows that DNA methylation is a plausible mediator of these mechanisms.

## Conclusions

Epigenetic modifications are at the interface between environment and genetics, creating a mechanism by which life experiences lead to long-lasting changes in gene expression. Here we show that the induction of peripheral nerve injury has an impact on the brain in the form of decreased DNA methylation in the PFC and amygdala 5–6 months following initial injury. In addition, these pathological changes can be attenuated with environmental enrichment, an intervention that ameliorates neuropathic pain in these animals. Furthermore, global methylation in the PFC correlates to symptom severity. Abnormal DNA methylation in the PFC may therefore provide a molecular substrate for pain-related dysfunction in brain structure and function. Targeting of these changes represents a potential novel therapeutic strategy for the treatment of chronic pain. The implications of epigenetic involvement in chronic pain are wide reaching and may alter the way we think about pain diagnosis, research and treatment.
